# Machine learning and microsimulation techniques on the prognosis of dementia: A systematic literature review

**DOI:** 10.1371/journal.pone.0179804

**Published:** 2017-06-29

**Authors:** Ana Luiza Dallora, Shahryar Eivazzadeh, Emilia Mendes, Johan Berglund, Peter Anderberg

**Affiliations:** 1 Department of Computer Science, Blekinge Institute of Technology, Karlskrona, Sweden; 2 Department of Health, Blekinge Institute of Technology, Karlskrona, Sweden; Banner Alzheimer's Institute, UNITED STATES

## Abstract

**Background:**

Dementia is a complex disorder characterized by poor outcomes for the patients and high costs of care. After decades of research little is known about its mechanisms. Having prognostic estimates about dementia can help researchers, patients and public entities in dealing with this disorder. Thus, health data, machine learning and microsimulation techniques could be employed in developing prognostic estimates for dementia.

**Objective:**

The goal of this paper is to present evidence on the state of the art of studies investigating and the prognosis of dementia using machine learning and microsimulation techniques.

**Method:**

To achieve our goal we carried out a systematic literature review, in which three large databases—Pubmed, Socups and Web of Science were searched to select studies that employed machine learning or microsimulation techniques for the prognosis of dementia. A single backward snowballing was done to identify further studies. A quality checklist was also employed to assess the quality of the evidence presented by the selected studies, and low quality studies were removed. Finally, data from the final set of studies were extracted in summary tables.

**Results:**

In total 37 papers were included. The data summary results showed that the current research is focused on the investigation of the patients with mild cognitive impairment that will evolve to Alzheimer’s disease, using machine learning techniques. Microsimulation studies were concerned with cost estimation and had a populational focus. Neuroimaging was the most commonly used variable.

**Conclusions:**

Prediction of conversion from MCI to AD is the dominant theme in the selected studies. Most studies used ML techniques on Neuroimaging data. Only a few data sources have been recruited by most studies and the ADNI database is the one most commonly used. Only two studies have investigated the prediction of epidemiological aspects of Dementia using either ML or MS techniques. Finally, care should be taken when interpreting the reported accuracy of ML techniques, given studies’ different contexts.

## Introduction

Dementia is a complex disorder that affects the brain. It is most prevalent in the elderly population, responsible for a progressive cognitive decline severe enough to interfere with the patient’s daily functioning and independence. Although decades of research have been dedicated to studying it, little is known about its mechanisms and there is still no disease modifying treatment that is able to stop or significantly delay its progression [1]. The most common form of dementia pathology is the accumulation of amyloid plaques in the brain and tau proteins inside the neurons. Amyloid plaques are very small in size (about 0.1 mm) and are formed by protein fragments Aβ, surrounded by dysfunctional neurons, whilst tau proteins accumulated inside the neurons form fibrillary tangles [2]. Together, these two factors are believed to be highly correlated to the neurodegeneration process [2].

Beyond the loss of independence, studies estimate that persons with dementia face mortality risks two times higher than that for similar groups without dementia [3] and deal with 2 to 8 additional chronic diseases that may accelerate their decline in daily functioning [1,4]. There are also consequences for the caregivers, especially for the family of the affected persons, who report low confidence in managing the condition, high levels of strain and depressive symptoms [5].

The demographic changes, with an increasing number of older people worldwide, will dramatically increase the cost in health and care programs. In 2011 the global estimated number of people with dementia was 35.6 million, and the trend points to a 100% increase within 20 years [6]. In comparison to other chronic disorders, in 2010 the global direct cost (prevention and treatment) and indirect cost (owing to mortality and morbidity) of cancer and diabetes were respectively $290 billion and $472 billion, while in 2014 the direct cost of Alzheimer’s Disease (AD), in USA alone, was of $214 billion [2].

Given that dementia is a serious disorder that brings so many challenges to patients, caregivers and public entities, and for which research on treatments are still on course, it is extremely important to investigate dementia’s prognosis. Prognostic estimates can aid researchers in finding patterns on disease progression, support public entities in allocating resources for the creation and maintenance of healthcare programs, and also aid patients and their caregivers in understanding more about their condition [7]. To be able to derive such useful estimates about dementia, reliable patient data is needed, like the ones from randomized clinical trials e.g. the Finnish Geriatric Intervention Study to Prevent Cognitive Impairment and Disability (FINGER), and the Healthy Aging Through Internet Counseling in the Elderly (HATICE), or other study initiatives/consortiums e.g. the Swedish National Study on Aging and Care (SNAC), Alzheimer's Disease Neuroimaging Initiative (ADNI), and European Alzheimer’s Disease Consortium Impact of Cholinergic Treatment Use (EADC-ICTUS).

The existence of health data allows for the execution of analyses that can derive several types of prognostic estimates. Two data analysis approaches that are specially focused in prediction and could be of great service to prognostic studies are: machine learning (ML) and microsimulation (MS) [8,9].

ML is already widely employed in biological domains such as genomics, proteomics, microarrays, systems biology, evolution and text mining [8]. It comprises a group of techniques that are able to learn from a set of examples (training set) to perform a task, so that this task can be performed with a completely new data set [10]. The most common learning approaches for ML techniques are supervised or unsupervised learning. When a supervised learning approach is used, the training set is composed by labeled examples (input and output variables). The most common tasks that use such approach are classification, in which the data is categorized in a finite number of classes; and regression, in which a function maps input variables to a desirable output variable [11]. Unsupervised learning happens when the data is not labeled, so the algorithm will work to find patterns that describe the data. Clustering is a common employed task, and characterizes the partitioning of a data set following a certain criteria [10]. Depending on the available health data and problem that needs to be solved, both supervised and unsupervised approaches can be used for prognostic estimates [11].

In the past there has been a number of studies that used standard statistics for disease prediction and prognosis (e.g. cancer, dementia). Such studies were feasible because “our dependency on macro-scale information (tumor, patient, population, and environmental data) generally kept the numbers of variables small enough so that standard statistical methods or even a physician’s own intuition could be used to predict cancer risks and outcomes” [12]. However, the world has changed into a reality where high-throughput diagnostic and imaging technologies are used, which, as a consequence lead to an overwhelming number of molecular, cellular and clinical parameters [12]. This has been the case in cancer as well as in dementia research, amongst other diseases. In such circumstances, as clearly stated by Cruz and Wishart [12] “human intuition and standard statistics don’t generally work. Instead we must increasingly rely on nontraditional, intensively computational approaches such as machine learning. The use of computers (and machine learning) in disease prediction and prognosis is part of a growing trend towards personalized, predictive medicine”. Such argument is also shared by others, such as Kourou et al. [10], who have even explicitly removed from their Mini review any studies that employed conventional statistical methods (e.g. chi-square, Cox regression). Finally, we also share the same view as Cruz and Wishart [12] with respect to the advantages that ML techniques provide, when compared to standard statistics: “Machine learning, like statistics, is used to analyze and interpret data. Unlike statistics, though, machine learning methods can employ Boolean logic (AND, OR, NOT), absolute conditionality (IF, THEN, ELSE), conditional probabilities (the probability of X given Y) and unconventional optimization strategies to model data or classify patterns”; further, ML “still draws heavily from statistics and probability, but it is fundamentally more powerful because it allows inferences or decisions to be made that could not otherwise be made using conventional statistical methodologies..”. [12]

One point to note is that the studies that use ML techniques for prognosis deal mostly with individuals as their unit of study. However, prognosis can also be extended beyond individuals to also include populations (e.g. studies by Suh and Shah [13] and Jagger et al [14]). To focus upon populations may be a suitable choice for example, when addressing the dementia family of diseases, as their long-term presence and considerable direct or indirect costs require significant investment, economic arrangements, and development of care facilities & infrastructure. Therefore, to address dementia prognosis in populations, we included MS methods, as this is a technique that has been traditionally used for prediction in populations.

MS models are closely related to an agent-based simulation model and aim to model individuals in a specific context though time [15,16]. The result of this simulation can give insights about the overall future of that population. MS has been used in healthcare to study how the screening programs can change morbidity and mortality rates or to estimate the economic aspects of diagnosis in specific diseases [9]. The same rationale could be applied in prognosis of dementia-related diseases and to apply MS as means to obtain insights on dementia prognostic isolation at the population level (in contrast to an individual level).

Given the abovementioned motivation, this paper aims to detail a systematic literature review that investigates the state of the art on how the ML and MS techniques are currently being applied to the development of prognostic estimates of dementia, aiming to answer the research question: “How are the machine learning and microsimulation techniques being employed by the researches on the prognosis of dementia and comorbidities?”.

This paper is organized as follows: the Method section presents the approach followed to conduct the review; the Results section presents summarized data from the included studies; the Discussion section argues about the results and presents threats to validity; and the Conclusion section presents final statements and comments on future work.

## Methods

A systematic literature review (SLR) identifies, evaluates and interprets a significant and representative sample of all of the pertinent primary studies of the literature concerning a topic of the research. SLRs execute a comprehensive search following a preset method that specifies focused research questions, criteria for the selection of studies and assessment of their quality, and forms to execute the data extraction and synthesis of results [17]. Among the motivations for conducting a SLR, the most common are: to summary all the evidence about a topic; to find gaps in the research; to provide a ground for a fundament to new research; and to examine how the current research supports a hypothesis. Performing a SLR comprises the following steps: (i) identify the need for performing the SLR; (ii) formulate research questions; (iii) execute a comprehensive search and selection of primary studies; (iv) assess the quality and extract data from the studies; (v) interpret the results; and (vi) report the SLR [18,19].

The SLR reported herein is part of a multidisciplinary project, in which five participants with different expertise (health, machine learning and bioinformatics) took part. Throughout the text, references to the authors will use a notation, in which A1 refers to the first author; A2 refers to the second author, and so forth.

The main research question this SLR aims to address is: “How are the machine learning and microsimulation techniques being employed by the researches on the prognosis of dementia and comorbidities?”. This main question was decomposed further into five research questions:

- RQ1: Which ML and MS techniques are being used in the dementia and comorbidities research?
- RQ2: What data characteristics (variables, determinants and indicators) are being considered when applying the ML or/and MS techniques (physiological, demographic/social, genetics, lifestyle etc)?
- RQ3: What are the goals of the studies that employ ML or MS techniques for prognosis of dementia and comorbidities?
- RQ4: How is data censoring being handled in the studies?
- RQ5: Do the studies focus on individuals or populations?

Partial results for questions RQ2 and RQ3 were the subject of a previous publication by the same authors [18]. The present paper builds upon these questions and additionally presents the results of the other two additional research questions.

Further, the key terms related to comorbidities were included in the search string to ensure that relevant studies about ML or MS for the prognosis of a disease, where dementia is considered a comorbidity to that disease would also be retrieved from the database searches, even when the term dementia was not mentioned in the paper’s title or abstract.

The protocol that guided the execution of this SLR is available at https://goo.gl/6Jddw3

### Search strategy

To address the research questions, a search string was defined using the PICO approach, which decomposes the main question into four parts: population, intervention, comparison and outcome [19]. The comparison component was discarded because the SLR was mainly concerned with a characterization. For each of the remaining components, keywords were derived and their rationale can be represented as follows:

- **Population**: Studies that present research on dementia and comorbidities. Dementia’s keywords were selected from the “Systematized Nomenclature of Medicine–Clinical Terms” and selected by A4. Comorbidities’ keywords were extracted from the Marengoni et al. SLR in this topic [20].
- **Intervention**: ML or MS techniques. The ML keywords were selected from the branch “Machine Learning Approaches” of the “2012 ACM Computing Classification System”. The MS keywords were selected by A2.
- **Outcome**: Prognosis on dementia and comorbidies. The prognosis keywords were provided by A4.

The automated searches were performed in the Pubmed, Web of Science and Scopus databases. Table 1 shows the search string used for the Pubmed automated search, but note that this search string was adapted to each of the other databases’ search context.

**Table 1:** Search string used in the Pubmed automated search.

Search Date	October 23^rd^ of 2015
("Dimentia" OR "Dementia" OR "Alzheimer" OR "Mixed Dementia" OR "Vascular Dementia" OR "Lewy Bodies" OR "Parkinson" OR "Creutzfeldt-Jakob" OR "Normal pressure hydrocephalus" OR "Huntington disease" OR "Wernicke-Korsakoff Syndrome" OR "Frontotemporal Dementia" OR "Neurosyphilis" OR "complex of Guam" OR "Subcortical leukoencephalopathy" OR "Comorbidities" OR "Comorbidity" OR "Co-morbidity" OR "multimorbidity" OR "multimorbidities" OR "multi-morbidity") AND ("Machine Learning" OR "Data Mining" OR "Decision Support System" OR "Clinical Support System") AND ("Classification" OR "Regression" OR "Kernel" OR "Support vector machines" OR "Gaussian process" OR "Neural networks" OR "Logical learning" OR "relational learning" OR "Inductive logic" OR "Statistical relational" OR "probabilistic graphical model" OR "Maximum likelihood" OR "Maximum entropy" OR "Maximum a posteriori" OR "Mixture model" OR "Latent variable model" OR "Bayesian network" OR "linear model" OR "Perceptron algorithm" OR "Factorization" OR "Factor analysis" OR "Principal component analysis" OR "Canonical correlation" OR "Latent Dirichlet allocation" OR "Rule learning" OR "Instance-based" OR "Markov" OR "Stochastic game" OR "Learning latent representation" OR "Deep belief network" OR "Bio-inspired approach" OR "Artificial life" OR "Evolvable hardware" OR "Genetic algorithm" OR "Genetic programming" OR "Evolutionary robotic" OR "Generative and developmental approaches" OR "microsimulation" OR "micro-simulation" OR "microanalytic simulation" OR "agent-based modeling") AND ("prognosis" OR "prognostic estimate" OR "predictor" OR "prediction" OR "model" OR "patterns" OR "diagnosis" OR "diagnostic" OR "Forecasting" OR "projection")	

### Study selection

The first step of the study selection was the execution of an evaluation round with 100 random papers from the 593 results returned from the automated searches. These had their title and abstracts assessed by A1, A2 and A3, according to inclusion and exclusion criteria defined previously in the protocol (see Table 2). This step was mainly concerned in maintaining the consistency of the selection between the participants throughout the SLR.

**Table 2:** Inclusion and exclusion criteria for assessing the studies returned by the searches.

Inclusion Criteria	Exclusion Criteria
Be a primary study in English; AND address research on dementia and comorbidities; AND address at least one ML or MS technique; AND address a prognosis related to dementia and comorbidities.	Be a secondary or tertiary study; OR be written in another language other than English; OR do not address a research on dementia and comorbidities; OR do not address at least one ML or MS technique; OR do not address a prognosis related to dementia and comorbidities.

The remaining 493 results had their title and abstracts assessed by A1 and A2, according to the inclusion and exclusion criteria. After the evaluations, 37 papers were selected. Then a one-iteration backward snowballing was carried out looking for possible additional studies. The 1199 new identified studies were assessed analogously as the previous ones, resulting in 41 new selected papers. Throughout the whole selection process, A3 and A4 acted in conflict resolution in the case where A1 and A2 couldn’t reach an agreement.

In total, 78 papers were selected to be fully read and assessed regarding its eligibility. The ones that successfully passed the established criteria previously defined in the protocol, had their relevant data extracted.

In order to minimize the chance of selecting studies with bias evidence, a quality assessment questionnaire was used. This questionnaire was adapted from Kitchenham’s guidelines [18] and can be found in the SLR protocol. If the grading attributed to a paper fell below 8 points (out of a total of 12), it would be rejected for quality reasons. The 8-point threshold was decided in the research group discussions involving all the authors. In this phase, a paper could also be rejected due to inclusion and exclusion criteria because the selection process adopted an inclusive approach. This means that during the reading of the titles and abstracts, in the case where the information provided was incomplete or too general it was selected to be fully read in the posterior phase. A common example is the case when the data analysis technique specified in the abstract was merely “classification”, so it was not possible to know if any machine learning occurred.

As in the study selection, a quality assessment evaluation round was performed beforehand to ensure consistency in the evaluations. A1, A2 and A5 participated in this task.

In total, 37 studies composed the final set of included primary studies and had their relevant data extracted, 7 papers were rejected due quality reasons, and 34 papers were rejected due to failing the inclusion and exclusion criteria. One reason for the high number of the latter was the decision to exclude the papers that used solely statistical methods as data analysis techniques to build the prognostic models.

The selected studies were also assessed for the risk of cumulative evidence bias. This was done by checking, in the case of the same research group with different studies in the final set of included primary studies, if it was justified having both studies (i.e different samples).

### Data collection

For the data collection, a base extraction form was defined in the protocol, but later in the study it was evolved based on the research group discussions. Table 3 lists and defines the collected variables.

**Table 3:** List of collected variables and their definitions.

Variable	Definition
**Conditions Studied**	For which dementia disorder is the study deriving a prognosis.
**Database used in the study**	Name and origin of the data source used to derive the prognosis of the studied dementia.
**Dataset Categories**	Classes in which the data units were divided into.
**Handling of censored data**	Description of the way in which censored data was handled.
**Follow-up Period**	Period of time, which the data units were followed.
**Data Analysis Techniques**	ML or MS techniques that were used to build the prognostic models.
**Model Variables**	The variables used in building the prognostic models.
**Aim of the Study**	The goal of the built prognostic models.
**Focus of the Study**	If the built prognostic models aim its predictions on an individual or population level.

In addition to these variables other basic data about the studies was collected, these were: title, authors, journal/source, year and type of publication. No summary measures were used.

Summary tables were used for the synthesis of results and no additional analyses were carried out.

## Results

**Fig 1:**
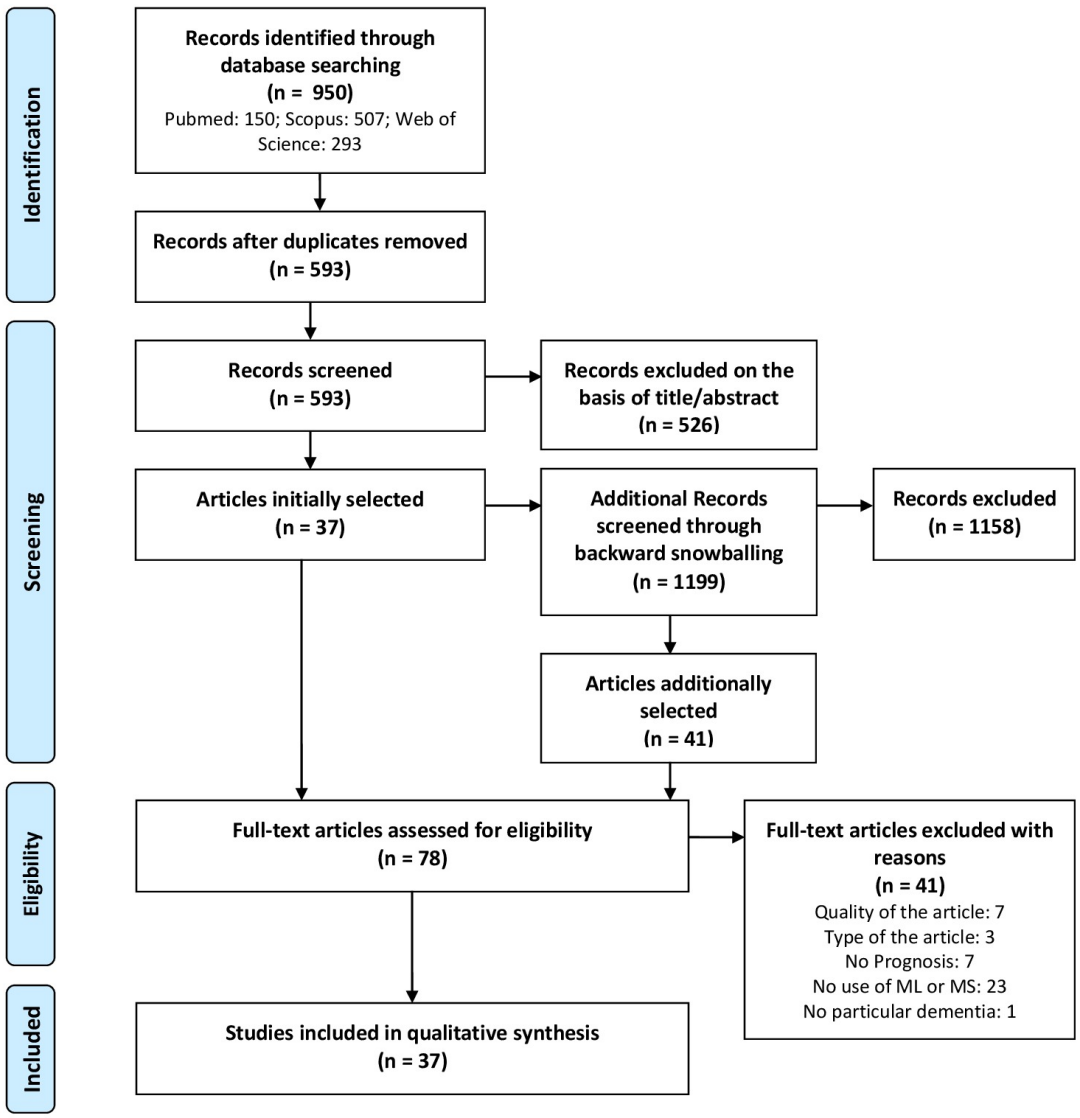
PRISMA flow chart.

The PRISMA (Preferred Reporting Items for Systematic Reviews and Meta-Analyses) Flow chart that describes the selection of the articles is shown in Fig 1.

A total of 78 results were assessed for eligibility having 37 studies selected as part of the final set of included primary studies, 7 studies excluded for falling out of the threshold of the quality assessment (8 out of 12), 3 studies excluded for not being a primary study, 7 studies excluded for not being about a prognostic estimate, 23 studies excluded for not making use of a ML or MS technique, and 1 study excluded for dealing with cognitive decline, but not dementia specifically (see S1 Table).

Three groups of common authors had 2 papers included in the final set of included studies, these were: Zhang, Daoqiang and Shen, Dinggang; Llano, Daniel A. and Devanarayan, Viswanath; and Moradi, Elaheh, Tohka, Jussi and Gaser, Christian. After the assessment for possible bias it was found that in these cases, either the sample varied or the categories of variables changed, not representing cumulative evidence bias to the SLR.

**Fig 2:**
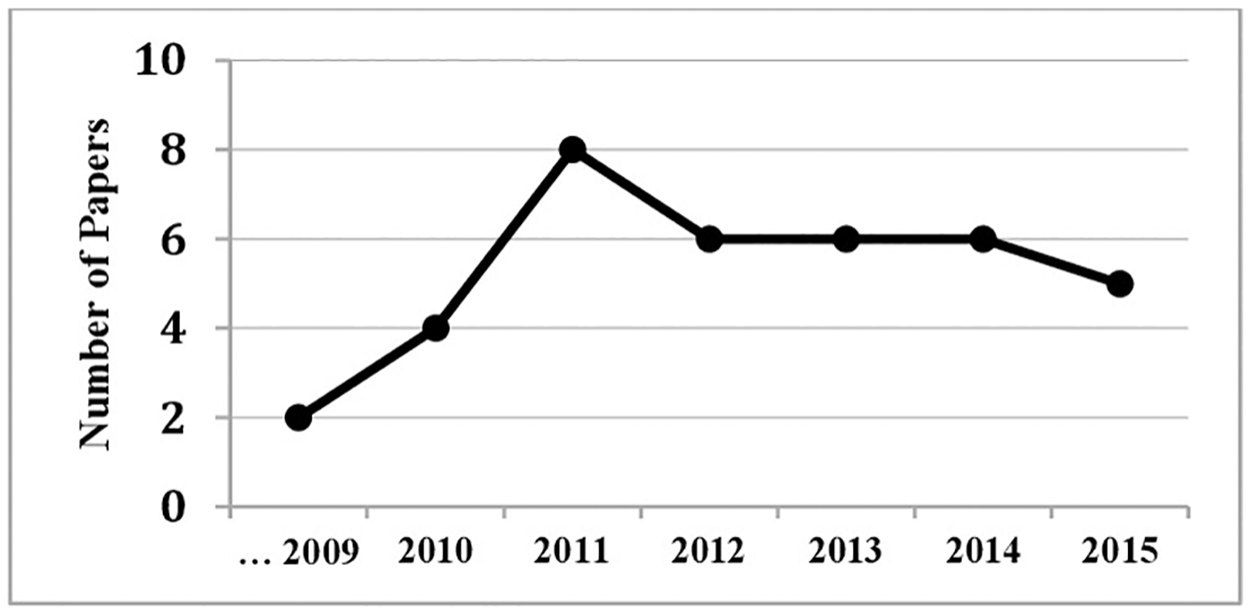
Frequency of published papers per year.

Fig 2 shows the frequency of primary studies per year of publication. It has to be remarked that the frequency showed for the year 2015 refers to studies published until October, when the search was performed.

### Identified machine learning techniques

This section presents the results that address research question RQ1: “Which ML and ML techniques are being used in the dementia and comorbidities research?”

Regarding the ML techniques, the synthesis of the extracted data shows that the most frequently used ML techniques were Support Vector Machines (SVM) (30 studies), Decision Trees (DT) (6 studies), Bayesian Networks (BN) (6 studies) and Artificial Neural Networks (ANN) (3 studies). These results are consistent with the cancer prognosis research, which also lists ANN, DT, SVM and BN as the ones most widely used [10]. In the cancer field, SVMs are relatively new algorithms that have been widely used due to its predictive performance, ANNs have being used extensively for almost 30 years; however the ideal ML technique to be used in a certain situation is dependent on the type of data to be used in the model, sample sizes, time constraints and the prediction outcome [11].

Other techniques that appeared less frequently are: Voting Feature Intervals (VFI), K-Nearest Neighbors (KNN), Nearest Shrunken Centroids (NSC) and Bagging (BA). These results will be explored in more detail next, so that firstly we provide a brief explanation of each ML technique, followed by a description on how it was applied for prognosis.

**Fig 3:**
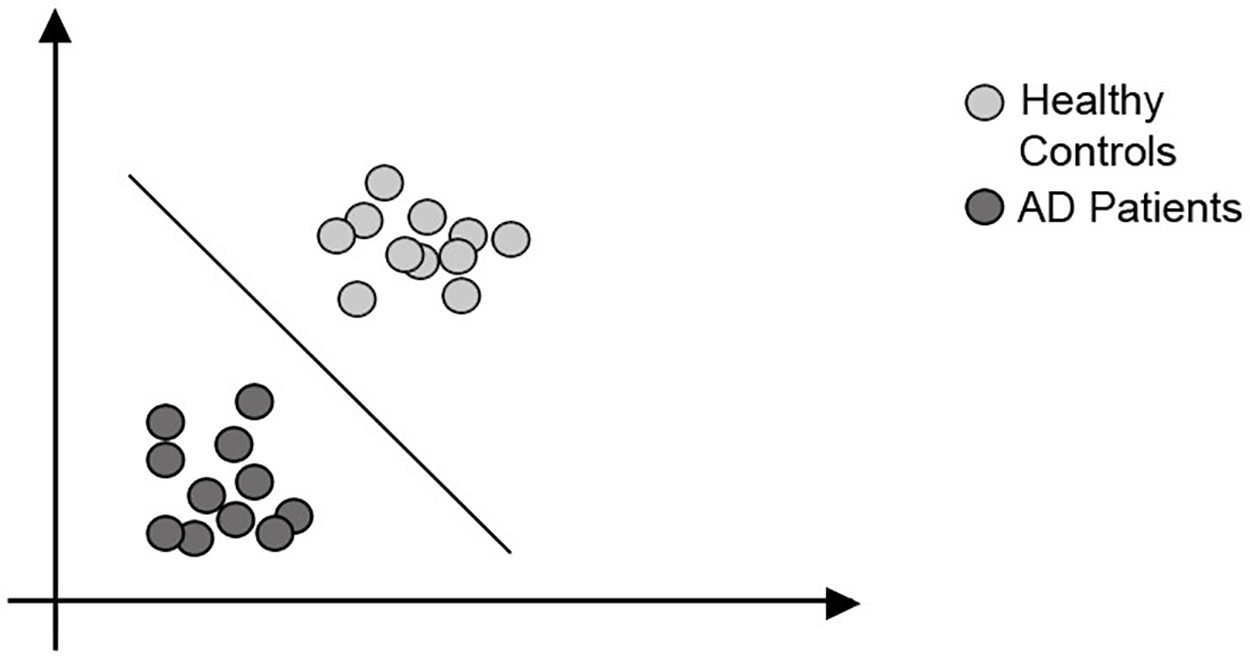
SVM classification example. The data points in the feature space are being classified in 2 classes.

Support Vector Machine (SVM) was originally proposed as an algorithm for classification problems; it is a relatively new technique compared to the other ML approaches. The classification process consists of mapping the data points (usually the study subjects) into a feature space composed of the variables that characterize these data points, except for the outcome variable. Then, the algorithm finds patterns in this feature space by defining the maximum separation between two or more classes, depending on the problem to be solved (see Fig 3) [21]. Contrary to some regression techniques, SVMs are not dependent on a pre-determined model for data fitting, although there are still algorithm specifications to be considered (e.g. choice of a kernel function) [22]; instead, it is a data-driven algorithm that can work relatively well in a scenario where sample sizes are small compared to the number of variables, reason why it has been widely employed by prognostic studies in tasks related to the automated classification of diseases [23].

Regarding the SLR results, SVMs were present in 30/37 selected studies, in 38 proposed models, and being by far the most used machine learning technique in the dementia prognosis research. These numbers account for the traditional SVM and variations (see Table 4). In all of the 30 selected studies the SVMs focused at binary classifications where the task was to discriminate mild cognitively impaired (MCI) patients that will or will not develop Alzheimer’s Disease (AD). In the general case, the problem is posed as either MCI converters versus MCI non-converters, or progressive MCI versus stable MCI classification. This outlines a situation in which a regression problem (“when will the MCI patients convert to AD?”) is formulated as a classification problem (“which MCI patients will convert to AD in X months?”) to be solved. Reasons for this could be due to limitations in the data used, i.e. the limited follow-up periods of the subjects included in the studies.

**Table 4:** Featuring studies that applied SVMs for the prognosis of dementia.

Variations of the Technique	Featuring Studies
Support Vector Machines	[24–45]
Radial Basis Function SVM	[38, 46–49]
Multi Kernel SVM	[35, 50–52]
Semi-supervised Low Density Separation	[40,48]
Domain Transfer SVM	[28]
Laplacian SVM	[28]
Relevance Vector Machines	[53]
SVM with a Logistic Regression Loss Function	[28]
Other proposed approaches to SVM	[28]

**Fig 4:**
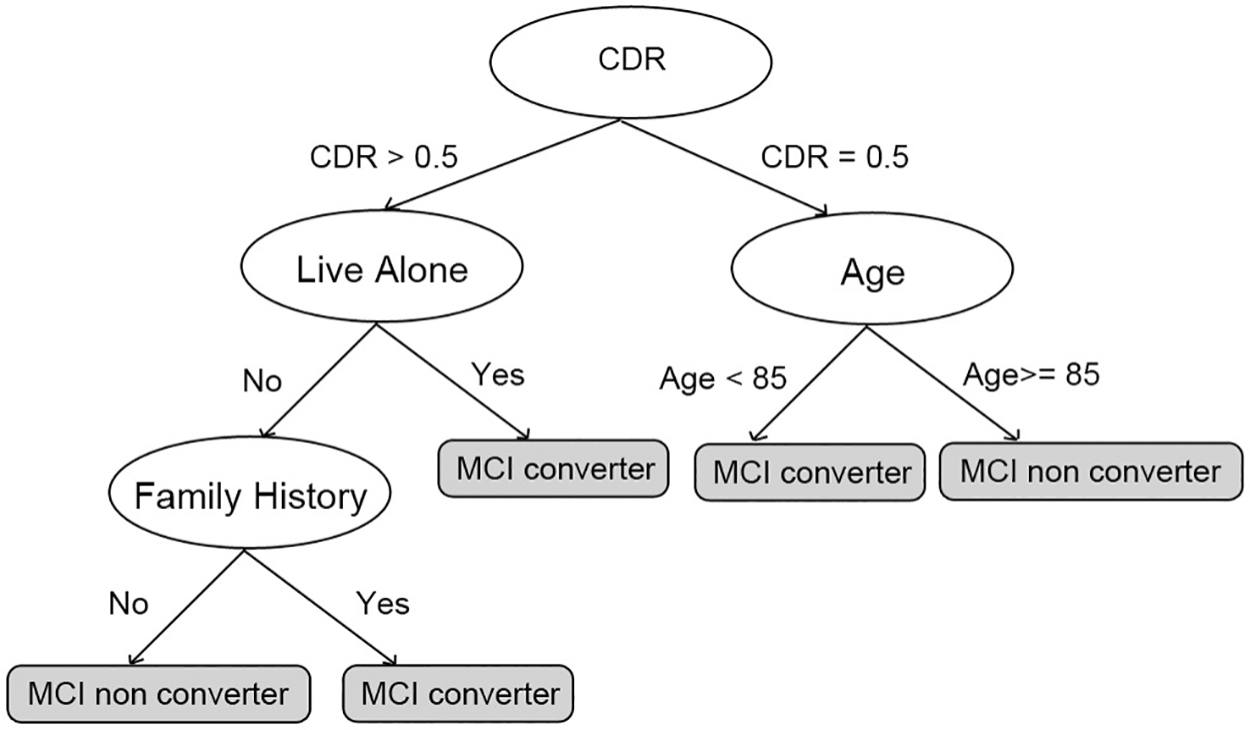
DT classification example. V(1–6) represent values that regulates the splits of the tree.

A **Decision Tree** (DT) is a classification algorithm in which the learned knowledge is represented in a tree structure that can be translated to if-then rules. DT’s learning process is recursive and starts by testing each input variable as to how well each of them, alone, can classify the labeled examples. The best one is selected as a root node for the tree and its descendant nodes are defined as the possible values (or relevant ratios) of the selected input variable. The training set is then classified between the descendant nodes according to the values of the selected input variable. This process is repeated recursively until no more splits in the tree are possible (see Fig 4) [54]. Like SVMs, DTs do not depend on a pre-defined model and are mostly used to find important interactions between variables. Being intuitive and easy to interpret, DTs have been used in prognostic studies as a tool for determining prognostic subgroups [55,12].

In this SLR, DTs were the second most frequently used ML technique, present in 6/37 selected studies and proposed in 7 models. The variations of this type of technique in the selected studies are shown in Table 5. It was employed for the same reason as SVM; except for one study that investigated the evolution of patients diagnosed with cognitive impairment no dementia (CIND) to AD.

**Table 5:** Featuring studies that applied DTs for the prognosis of dementia.

Variations of the Technique	Featuring Studies
Random Forests	[30, 38, 47, 56]
Decision Trees	[25, 57]
Boosted Trees	[30]

**Fig 5:**
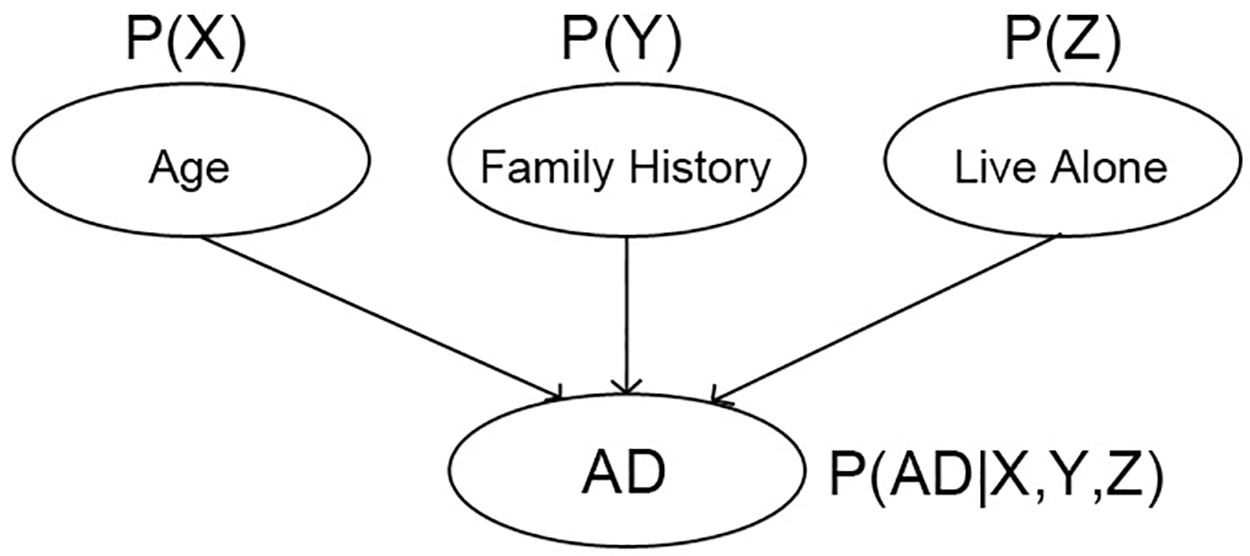
BN example. P(X-Z) represent probabilities and P(X-Z|X,Y,Z) represent conditional probabilities.

**Bayesian Networks** (BN) are directed acyclic graphs, in which each node represents a variable and each edge represents a probabilistic dependency. This structure is useful for computing the conditional probability of a node, given the values of the other variables or events. In a BN, the learning process is composed of two tasks: learning the structure of the graph and learning the conditional probability distribution for each node (see Fig 5) [58]. In this way, the classification in a BN estimates the posterior probability of a data point to belong to a class, given a set of features. BNs have been applied in the research for classification, knowledge representation and reasoning; however contrary to the other mentioned algorithms, BNs generally produce probabilistic estimations, rather than predictions per se [58]. A great advantage of BN in comparison to other techniques, such as ANNs and SVMs, which renders it benefits for its use in prognostic models, is that they do not require the availability of large amounts of data and can also encode the knowledge of domain experts [59]. However, a drawback in this technique is that it may not be expandable to a large number of features [60].

BNs were the second mostly used ML technique, along with DTs, present in 6/37 selected studies. The identified variations of BN algorithms are shown in Table 6. As previously mentioned, BN models were built for use in classification tasks related to the evolution of patients with MCI to Alzheimer’s, with the exception of one study that used BNs for events-based modeling of the progression to AD.

**Table 6:** Featuring studies that applied BNs for the prognosis of dementia.

Variations of the Technique	Featuring Studies
Naïve Bayes	[30, 42]
Gaussian Naive Bayes	[27]
Markov Chains Monte Carlo	[61]
Bayesian Outcome prediction with Ensemble Learning	[62]
Gaussian Process Classification	[45]

**Fig 6:**
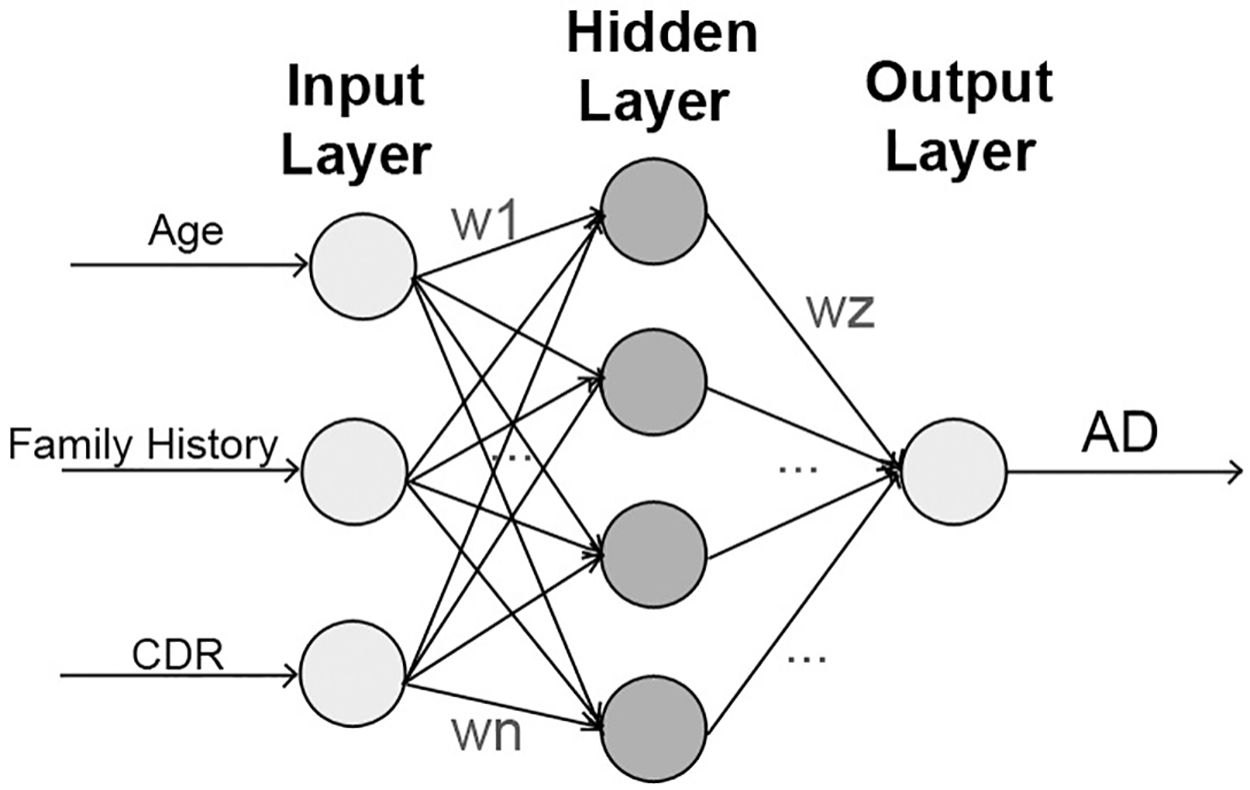
ANN example. The weights of the edges are represented by w(1-n).

An **Artificial Neural Network** (ANN) is a methodology that performs multifactorial analyses, which is desirable in the health area as medical decision-making problems are usually dependent of many factors. An ANN is composed of nodes connected by weighted edges in a multi-layer architecture that comprises: an input layer, one or more hidden layers and an output layer (see Fig 6). In the training process, inputs and outputs values are known to the network, while the weights are incrementally adjusted so that the outputs of the network are approximate to the known outputs [63]. Despite being a powerful predictor, ANNs are ‘black boxes’, which means that they are not able to explain their predictions in an intuitive way, contrary to DTs or BNs. Also, they require the specification of the architecture to be used beforehand (i.e. the number of hidden layers) [64].

ANNs were present in 3/37 of the selected studies and proposed in 4 models. The identified variations of the standard ANN in the selected studies are shown in Table 7. As was the case with all previous techniques, two studies aimed to predict the development of AD in patients with MCI and one aimed to predict the stage of AD on patients according to their cognitive measures.

**Table 7:** Featuring studies that applied ANNs for the prognosis of dementia.

Variations of the Technique	Featuring Studies
Artificial Neural Networks	[25,47,65]
Mixed Effects ANN	[65]

**Fig 7:**
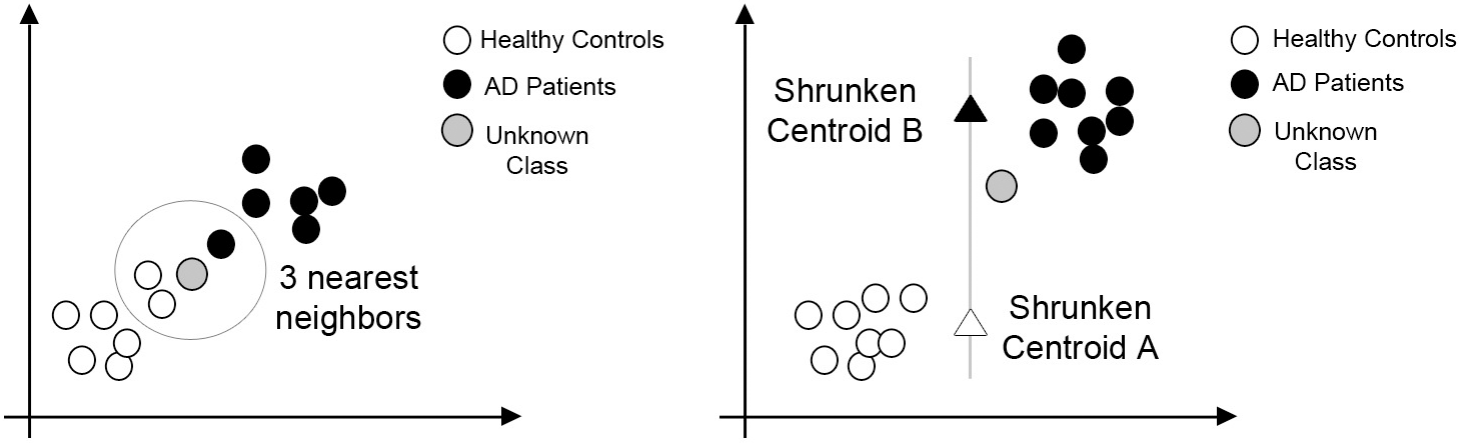
KNN (3-NN) and NSC examples. Both cases classify the unknown data point between 2 classes.

**K Nearest Neighbors (KNN)** is a classification algorithm that takes a data point from an unknown class and assigns it as an input vector in the feature space. Then, the classification process follows by assigning the unknown class data point to the class in which the majority of the K nearest data points belong to (see Fig 7) [66]. The distance between data points is usually measured by Euclidean distance, but it is possible to employ other measures. KNN is one of the simplest ML classification algorithms and have been used in a wide range of applications; however, it can be computationally expensive in a highly dimensional scenario. Further, it considers all features to be equally weighted, which can be a problem if the data has superfluous attributes [60].

The **Nearest Shrunken Centroids (NSC)** classification process starts by calculating the centroids to each of the classes that an unknown data point could belong to. In this context, the centroids are the mean feature vectors of each of the possible classes. Then, the algorithm shrinks the centroids toward the global centroid by a certain threshold [67]. This shrinkage operation acts as a form of feature selection, as it eliminates from the prediction rule, the components of the centroid that are equal to the correspondent component of the overall centroid [68]. Then, the algorithm sets the unknown data point to the class that has the shortest distance to its shrunken centroid (see Fig 7). As in KNN, the distance measure can be Euclidean or other. In the medical field, this algorithm was proposed to deal with the problem of predicting a diagnostic category by DNA microarrays, being useful in a high dimensionality scenario; yet a disadvantage of NSC is the arbitrary choice of shrinkage threshold [67].

**Voting Feature Intervals (VFI)** is an algorithm with a classification process similar to the BN, but instead of assigning probabilities to each possible class, VFI assign votes between feature intervals among the classes. The classification output is the class with the highest sum of votes [69]. One downside of this algorithm is that it is best applicable to contexts where the features are considered independent of each other, which may not always be the case [69]. On the other hand, the VFI algorithm can perform well in scenarios that may have many superfluous features to the classification task, which is also the reason why it was employed in the prognosis study [41].

**Bagging (BA)** or Bootstrap Aggregation is an ensemble ML technique. This means that it is actually a predictor created from an aggregation of different predictors. It uses bootstrapping to replicate the data set in new data sets that are used to make new predictions. In a classification task, different predictors will assign an unknown data point to a class. Then, it choses class it was classified in the most cases. BA is a method that is useful in the case of instable predictors to reduce the variance and prevent overfitting [70].

The studies that featured KNN, NSC, VFI and BA are shown in the Table 8. In all of these cases, studies aimed to predict the MCI evolution to AD.

**Table 8:** Featuring studies that applied other machine learning techniques for the prognosis of dementia.

Variations of the Technique	Featuring Studies
K Nearest Neighbors	[30,47]
Bagging	[47]
Nearest Shrunken Centroids	[30]
Voting Feature Intervals	[42]

### Identified microsimulation techniques

This section presents the results for RQ1 that relate to MS techniques.

In a typical MS model, a database of samples from a population exists. Each record in the database represents an individual and their associated states. Thus, in each time-step of the simulation a record at a time is being updated by applying a collection of rules [16]. The updated database at each time step shows the course and trajectory of changes in the population and therefore aggregative indicators can be extracted from this database. MS models contrast with other aggregative simulation models in the way they represent individuals in a population rather than aggregative variables and collective representations. Further, MS differ from agent-based simulations, as the focus of the first is on the trajectory and independent reaction of each individual unit and it is assumed that the units are independent [9, 16].

In the selected studies in our SLR, two papers have used MS techniques: Furiak et al. [71] through a simulation of **Time-to-Event (TTE)** for individuals; and Stallard et al. [72] through a **Grade of Membership (GM)** approach.

Furiak et al. [71] uses TTE to simulate the impact of future hypothetical screening and treatment interventions in delaying AD in a specific population. Stallard et al. [72] applies a GM approach to represent a multidimensional multi-attribute model of AD progress [72]. The term "microsimulation" is not used in this study; however, approaches similar to microsimulation were applied. In this study the impact of a future hypothetical successful intervention in slowing AD progress rate on MEDICARE and MEDICAID programs in the USA has been simulated by aggregating predictions on individual levels.

Any of these studies can be an example of instantiation of the MS approach in population level prognosis. For example in the study by Furiak et al. [71], a baseline population of individuals was created according to the relevant incident data from available studies. Each simulated individual, a record in a table, goes through a risk of developing AD and then after is being exposed to a time-to-event of being diagnosed as AD through screening (in different strategies). The diagnosed simulated individuals delay their progress to AD by receiving a hypothetical treatment. The aggregated values of individual progressions toward AD can be compared to real world situation in order to have an understanding of the performance of different screening strategies in the presence of a possible delaying treatment.

### Data characteristics used in the models

Table 9 shows the summary of data regarding RQ2: “What data characteristics (variables, determinants and indicators) are being considered when applying the ML or and MS techniques (physiological, demographic/social, genetics, lifestyle etc)?”

**Table 9:** Identified data characteristics in the included studies.

Variable Category	Variable Subcategory	Number of Studies	Featuring Studies
**Neuroimaging:** 28 ML studies	**MRI**	27	[24–26, 28, 31–37, 39–46, 48–53, 61, 62]
	**PET**	8	[27, 28, 33, 35, 45, 50–52]
**Cognitive Measures:** 5 ML studies, 1 MS study	**MMSE**	2	[34, 65]
	**ADAS-cog**	2	[38, 56]
	**Other (CDR, FAQ, Buschke Cued Recall)**	3	[32, 57, 65]
**Genetic:** 5 ML studies	**ApoE**	5	[33, 38, 41, 44, 45]
	**Family History**	1	[41]
**Lab Test:** 10 ML studies	**CSF**	8	[28, 30, 32, 33, 35, 45, 45, 51]
	**Other Lab tests**	4	[38, 44, 45, 47]
**Demographic:** 5 ML studies, 2 MS studies	**Age**	4	[30, 40, 53, 65]
	**Other demographic**	3	[38, 71, 72]

**Abbreviations: MRI**: Magnetic Resonance Imaging; **PET**: Positron Emission Tomography; **MMSE**: Mini Mental State Examination; **ADAS-cog**: Alzheimer's Disease Assessment Scale-cognitive subscale; **CDR**: Clinical Dementia Rating; **FAQ**: Functional Activities Questionnaire; **CSF**: Cerebrospinal Fluid

ML methods try to learn the relationship between a set of variables, i.e. variates, and the result variable, i.e. covariate. The studies in our collection used variables as variates while they were focused mostly on a binary prognosis variable (usually indicating development/no development to AD). This binary variable was accompanied with degrees of MMSE (Mini Mental State Examination) and ADAS-cog (Alzheimer's Disease Assessment Scale-cognitive subscale) cognitive scores in the study by Zhang *et al*. [52].

Table 9 also summarizes the connection between variables and studies. Note that variables that were considered in a study but did not contribute to its final result are not shown. Variables were categorized as neuroimaging, cognitive measures (neuropsychological), genetic, lab test, and demographic. Regarding the neuroimaging variables, in all of the included studies, automatic feature extraction techniques were used, either in the form of a software tool (e.g. FreeSurfer for MRI scans) or via their own ML technique being applied to create the models. A reason for this could be the interest in the implementation of more automated methods for identifying the development of AD in MCI patients.

Further, studies that examined more than one variable contributed more than once to the subcategories, while their contribution to categories was counted only once; therefore, the number shown for categories might be smaller than the total sum of numbers for their subcategories. Also, the smaller subcategories were grouped together. Finally, for the two MS studies, demographic and cognitive scores variables were considered as the input variables.

### Goals of the prognosis studies on dementia

Table 10 shows the summary data concerning the RQ3: “What are the goals of the studies that employ ML or MS techniques for prognosis of dementia and comorbidities?”.

**Table 10:** Goals of the studies in respect to the prognosis of dementia.

Study Goals	Count	Conditions Studied	Type of Data Analysis	Featuring Studies
Predict the development of Alzheimer’s Disease from Mild Cognitive Impairment	32	Alzheimer's Disease, Mild Cognitive Impairment	Machine Learning	[24–53, 56, 62]
Predict the development of Alzheimer’s Disease from Cognitive Impairment No Dementia	1	Alzheimer's Disease, Cognitive Impairment No Dementia	Machine Learning	[57]
Model disease stage through Mini Mental State Examination score	1	Alzheimer's Disease	Machine Learning	[65]
Events-based disease progression modeling	1	Alzheimer's Disease, Huntington's Disease	Machine Learning	[61]
Estimate the clinical course of mild Alzheimer’s Disease to Alzheimer’s Disease to death, and estimate costs (MEDICARE and MEDICAID)	1	Alzheimer's Disease, Mild Cognitive Impairment	Microsimulation	[72]
Evaluate screening and treatment to delay Alzheimer’s Disease	1	Alzheimer's Disease	Microsimulation	[71]

The aggregated data shows that the majority of studies, 32/37, intended to investigate the progression of MCI to AD, posing the problem as either MCI converters versus MCI non-converters, or progressive MCI versus stable MCI. These studies identified the need for the AD prognosis and proposed models for its prediction, usually within 6 to 36 months until development. Exceptions are Adaszewski *et al*. [24], and Chen *et al*. [62], which constructed models for 48 and 60 months prediction, respectively. Most of these studies used data from the Alzheimer’s Disease Neuroimaging Initiative (ADNI) in their models, what could explain the limitation of the prediction time as the follow-up period is limited.

A variation of this goal was the prediction of AD, but from Cognitive Impairment No Dementia, instead of MCI. This study [57] was conducted using data from the Canadian Study of Health and Aging (CSHA).

The goal of Tandon *et al*. [65] was the use of patients’ longitudinal data of the Layton Aging and Alzheimer’s Research Center (LAARC) to model the time-course of AD in terms of their cognitive functions. The chosen variable was the MMSE score (Mini Mental State Examination). Likewise, Fonteijn *et al*. [61] also had disease progression modeling as the goal, but in this study the model is characterized as an events-based model. Two types of events were considered: transitions to later clinical status (i.e. presymptomatic AD to MCI) and atrophy events measured by MRI scans. This is also the only study that approached a dementia disorder other than AD, building models for both AD, and Huntington’s Disease.

Stallard *et al*. [72] explored disease progression through MS aiming the estimation and comparison of costs (MEDICARE and MEDICAID) for slowing AD advancement in both patients with mild AD and moderate AD. Finally, Furiak *et al*. [71], applied MS to provide a framework for the screening of AD, which investigated treatment interventions for delaying the AD progression in a population.

### Handling of censored data

Data censoring happens when the information about the individual time to the event of interest is unknown for some participants [73]. This can occur when information on the time to event is unavailable due to loss to follow-up or due to the non-occurrence of the outcome event before the trial end [73][74]. In this SLR, only two out of the 37 selected studies addressed censored data, explicitly. The Craig-Schapiro et al. study [30] used Cox proportional hazard models (CPHM) to assess which baseline biomarkers should be considered in the ML multivariate models targeting at their ability to predict the conversion from cognitive normalcy (CDR 0) to very mild or mild dementia (CDR 0.5 and 1). They stated that participants who did not develop very mild or mild dementia during the follow-up were statistically censored.

In the Plant et al. study [42] data censoring is addressed as a threat to validity, arguing that for shorter follow-ups (30 months in this study), there may be patients classified as MCI with a MRI pathological pattern who had not yet developed AD during the follow-up.

The remaining studies included herein did not make any explicit statements about data censoring. Note that the studies by Escudero et al. [33], Moradi et al. [40] and Gaser et al. [53] performed a survival analysis to estimate a hazard ratio for the MCI conversion to AD using CPHM, which is a technique that is able to deal with censored data; however, no specific remark about data censoring was made.

### Focus of the studies

The last research question of this SLR (RQ5) was: “Do the studies focus on individuals or populations?”.

Out of the 37 included studies, only the two studies that used the MS methods [71,72] focused on populations and the rest of papers, all using ML, focused on the prognosis of dementia in an individual level.

## Discussion

### Discussion of the current evidence

The main findings from this SLR are summarized in the following points: (i) most of the research is focused on the use of neuroimaging for predicting the development of AD from MCI, using the ADNI database; (ii) estimations are usually made up to 36 months before the development to AD; (iii) lifestyle and socioeconomic variables were absent in the assessed models; (iv) data censoring is not addressed in the vast majority of the studies included in our SLR; (v) the focus of the research is mostly on individual level.

There is an indication that North America is leading the research on treatments for the preclinical stage of AD whilst Europe leads the lifestyle interventions for the prevention of dementia [2]. In what concerns studies that make use of high-level computational techniques, such as ML and MS, the findings of this SLR are consistent with the first part of this sentence.

Most of the research dedicated to the prognosis of dementia and that make use of ML techniques is focused on using neuroimaging to predict the development of AD from MCI, particularly making use of the ADNI database. A consequence of this scenario is the almost exclusive focus of the recent research in validating biomarkers to be used in treatment trials, since this is the overall objective of ADNI [75]. This intensive research on biomarkers is important to make the pharmaceutical research faster and to reduce the exposure of ineffective experimental drugs [76].

Another important aspect regarding the overall preference for the neuroimaging variables, throughout the included studies in this SLR, is that most of the prognosis research is concerned with a single aspect of dementia, and prognostic estimates should consider a multivariable approach. The reason for that is the variability among patients that could make the single predictor variable not very effective [77]. Furthermore, ADNI does not include in its database subjects with important comorbidities, considering dementia and the elderly population, like cancer and heart failure [75]. Additionally, as ADNI is not an epidemiologic study, there is a risk that the utility of methods used in the studies would be tailored to the ADNI’s specific conditions.

Still on the topic of the development of AD in persons with MCI, another important aspect to be discussed is how much time beforehand the proposed models in the current research can predict this conversion. The majority of them are set out to do this task in a period of up to 36 months. Putting aside the accuracy aspect of these predictions (which of course is of great importance), would this time constraint be enough to employ preventive strategies (pharmaceutical or non-pharmaceutical) on screened patients so to delay their progression to AD? An important consideration in the research for treatments is that they prolong the time patients spend in the most amenable stages of dementia and shorten the time they stay in the most severe stages, in which they suffer from a very low quality of life and when care is most costly [2].

The absence of studies using lifestyle and socioeconomic variables in models could point out a gap in the current research for more holistic approaches to the prognosis of dementia. Another possibility is that studies that are investigating these may simply apply other data analysis methods in deriving their predictions that are not ML or MS.

The fact that the handling of censored data was not made clear in all, except for two studies, can raise some concerns, as in most of the studies’ demographics the participants were divided into classes of normal controls, AD patients, MCI converters and MCI non-converters. The class of MCI non-converters could entail the case when a participant did not experience the event in evidence during the follow-up, characterizing a right-censoring scenario.

Lastly, with regard to the focus of the primary studies, the lack of studies that utilize MS for prognosis of dementia in individuals can be due to this method being usually applied to populations (rather than individuals), and also due to this method be based on simulating masses of individuals. However, in relation to ML methods, the lack of studies focusing on predicting the epidemiology of dementia in populations can be interpreted as a study gap.

### Methodological issues

When discussing the techniques being used to derive prognostic estimates for dementia, one interesting aspect to note is the comparison between them, in relation to which one(s) performed best. This task proved challenging. Reasons for that are due to several limitations in interpreting such results, detailed next.

Firstly, the studies have used different validation procedures and this can make their comparison difficult. Even in the studies that used the same method for accuracy calculation, the difference in some parameters (e.g. the number of folds in cross validation) or how they calculated distance of a prediction to the test case could have an impact on the reported accuracy.

Further on, the reports on accuracy are based on different datasets, and for those who share the same dataset (such as ADNI) each might use a different number of records, which can impact the reported accuracy. Also, the majority of studies have considered MRI or PET images, where the quality of images or the image pre-processing applied before the ML method can impact their reported accuracy.

Regardless of the applied method, different variables have different predictive powers and when two papers that have used the same method report different variables, they should be compared considering these reported variables. Accuracy and other related indicators have their values compared to a golden standard that determines the existence of AD (or any other progression). In some studies they have chosen other indicators rather than the golden standard (i.e. cerebral biopsy/autopsy versus CDR/GDS/ADL/ADAS-cog/MMSE numbers). Lastly, the follow-up period is different in the studies and maybe longer follow-ups would result in higher sensitivity reports.

Further, there are three commonly used types of accuracy that can be attributed to prognosis models: discrimination, calibration, and reclassification [78]. The discriminatory accuracy is the ability of the prognosis model in separating individuals regarding the outcome, while the calibration accuracy is how much the prognosis model risk prediction complies with observed risks in a population. In reclassification, one is interested in measuring the added predictive ability by a new biomarker [79,80]. With regard to the primary studies in this SLR, they only reported the discriminatory accuracy of prognosis. The overall accuracy was the index most commonly reported (see S1 Table). Alongside that, in 18 studies the AUC was indicated. The predications mostly concern a binary discrimination between converting and non-converting MCI, while in one of the studies (Zhang et al. [52]) predication of MMSE and ADAS-cog scores was considered. It is noteworthy that the presentation of prognosis accuracy in this SLR’s primary studies contrasts with similar prediction studies in the cancer field [81].

### Limitations

Regarding the limitations of this SLR it can be addressed the issue of whether a suitable large representative sample of all the possible relevant primary studies were included in the final set, and also the non-medical background of the two researchers on the study team, who screened most of the papers (A1 and A2). To mitigate the first issue a more inclusive selection strategy has been taken. This means that in papers in which there were poor indications of the inclusive criteria in their title or abstract, the content of study was further investigated for a possible inclusion. For the second issue, if it was not clear the fit of the paper for prognosis, a member of research team with medical education background (A4) was consulted.

### Future perspectives

The results of this SLR presented research trends and gaps that should be addressed in future research on the prognosis of dementia. Based on these findings, further research should explore different combinations of ML and MS techniques, using a multivariable approach that includes the identified data characteristics as well as lifestyle and social factors.

## Conclusion

Through the SLR, 37 studies that focus on the prognosis of dementia by using ML or MS techniques were selected. These studies were summarized in terms of different aspects including types of techniques, variables or goals and focus of the studies.

Our findings pointed out that most of the studies were concerned about predicting the development of AD in individuals with MCI using one of ML techniques. Neuroimaging data was the most common data to be fed into ML techniques. Only two studies focused on prediction regarding populations, and those were the only two studies that applied MS techniques. We identified only a limited number of datasets are being used in the studies (most notably, the ADNI database).

## Supporting information

S1 Table Final set of included studies. (PDF)

S2 Table PRISMA checklist. (PDF)
