# Supplementary material for: Machine learning and microsimulation techniques on the prognosis of dementia: A systematic literature review
Source: PLoS One. 2017 Jun 29;12(6):e0179804. doi: 10.1371/journal.pone.0179804 (PMC5491044; doi:10.1371/journal.pone.0179804)
Supplement: S1 Table — (PDF) [file pone.0179804.s001.pdf]

Title	Study	Conditions Studied	Database Name	Data Analysis Technique	Best Accuracy Achieved
Automated hippocampal shape analysis predicts the onset of dementia in mild cognitive impairment	Costafreda, S. et al. [29]	AD, MCI	AddNeuroMed	ML	85.0%
Different multivariate techniques for automated classification of MRI data in Alzheimer's disease and mild cognitive impairment	Aguilar, C. et al. [25]	AD, MCI	AddNeuroMed	ML	84.9%
An MRI-derived definition of MCI-to-AD conversion for long-term, automatic prognosis of MCI patients	Aksu, Y. et al. [26]	AD, MCI	ADNI	ML	83.00%
Discriminant analysis of longitudinal cortical thickness changes in Alzheimer's disease using dynamic and network features	Li, Y. et al. [39]	AD, MCI	ADNI	ML	81.7%
BrainAGE in Mild Cognitive Impaired Patients: Predicting the Conversion to Alzheimer's Disease	Gaser, C. et al. [53]	AD, MCI	ADNI	ML	81.0%
Domain Transfer Learning for MCI Conversion Prediction	Cheng, B. et al. [28]	AD, MCI	ADNI	ML	79.4%
Predicting future clinical changes of MCI patients using longitudinal and multimodal biomarkers	Zhang, D. et al. [52]	AD, MCI	ADNI	ML	78.4%
Deep learning-based feature representation for AD/MCI classification	Suk, Heung-Il; Shen, Dinggang [51]	AD, MCI	ADNI	ML	75.80%
Anatomically constrained weak classifier fusion for early detection of Alzheimer's disease	Komlagan, M. et al. [37]	AD, MCI	ADNI	ML	75.6%
Hierarchical interactions model for predicting Mild Cognitive Impairment (MCI) to Alzheimer's Disease (AD) conversion	Li, H. et al. [38]	AD, MCI	ADNI	ML	74.76%
Machine learning framework for early MRI-based Alzheimer's conversion prediction in MCI subjects	Moradi, E. et al. [40]	AD, MCI	ADNI	ML	74.74%
Automatic Prediction of Conversion from Mild Cognitive Impairment to Probable Alzheimer's Disease using Structural Magnetic Resonance Imaging	Nho, K. et al. [41]	AD, MCI	ADNI	ML	72.3%

Manifold population modeling as a neuro-imaging biomarker: application to ADNI and ADNI-GO	Guerrero, R. et al. [34]	AD, MCI	ADNI	ML	71.0%
Bioprofile analysis: A new approach for the analysis of biomedical data in Alzheimer's disease	Escudero, J. et al. [33]	AD, MCI	ADNI	ML	68.2%
Inter-modality relationship constrained multi-modality multi-task feature selection for Alzheimer's Disease and mild cognitive impairment identification	Liu, F. et al. [50]	AD, MCI	ADNI	ML	67.83%
Identification of conversion from mild cognitive impairment to Alzheimer's disease using multivariate predictors	Cui, Y. et al. [32]	AD, MCI	ADNI	ML	67.13%
Accurate multimodal probabilistic prediction of conversion to Alzheimer's disease in patients with mild cognitive impairment	Young, J. et al. [45]	AD, MCI	ADNI	ML	66.7%
Magnetic resonance imaging biomarkers for the early diagnosis of Alzheimer's disease: a machine learning approach	Salvatore, C. et al. [43]	AD, MCI	ADNI	ML	66.0%
How early can we predict Alzheimer's disease using computational anatomy?	Adaszewski, S. et al. [24]	AD, MCI	ADNI	ML	63.7%
Evaluation of Plasma Proteomic Data for Alzheimer Disease State Classification and for the Prediction of Progression From Mild Cognitive Impairment to Alzheimer Disease	Llano, D. et al. [47]	AD, MCI	ADNI	ML	62.0%
Automatic classification of patients with Alzheimer's disease from structural MRI: a comparison of ten methods using the ADNI database	Cuingnet, R. et al. [31]	AD, MCI	ADNI	ML	-
Semi-supervised learning in MCI-to-ad conversion prediction - " When is unlabeled data useful?"	Moradi, E. et al. [48]	AD, MCI	ADNI	ML	-
Sparse learning and stability selection for predicting MCI to AD conversion using baseline ADNI data	Ye, J. et al. [44]	AD, MCI	ADNI	ML	-
Predictive markers for AD in a multi-modality framework: an analysis of MCI progression in the ADNI population	Hinrichs, C. et al. [35]	AD, MCI	ADNI	ML	-
Predicting conversion from MCI to AD with FDG-PET brain images at different prodromal	Cabral, C. et al. stages [27]	AD, MCI	ADNI	ML	-

Derivation of a new ADAS-cog composite using tree-based multivariate analysis: Prediction of conversion from mild cognitive impairment to alzheimer disease	Llano, D. et al. [56]	AD, MCI	ADNI	ML	-
Applying Automated MR-Based Diagnostic Methods to the Memory Clinic: A Prospective Study	Kloeppel, S. et al. [36]	AD, MCI	ADNI, Freiburg	ML	65.0%
Prediction of conversion from mild cognitive impairment to Alzheimer disease based on bayesian data mining with ensemble learning	Chen, R. et al. [62]	AD, MCI	ADNI, PCD	ML	81%
Automated detection of brain atrophy patterns based on MRI for the prediction of Alzheimer's disease	Plant, C. et al. [42]	AD, MCI	Clinic of Psychiatry at the Ludwig Maximilian University of Munich	ML	95.83%
An event-based model for disease progression and its application in familial Alzheimer's disease and Huntington's disease	Fonteijn, H.M. et al. [61]	AD, HD	Cognitive Disorders Clinic at the National Hospital for Neurology and Neurosurgery, Multidisciplinary HD Clinic at the National Hospital for Neurology and Neurosurgery	ML	-
Clinical Decision Trees for Predicting Conversion from Cognitive Impairment No Dementia (CIND) to Dementia in a Longitudinal Population-Based Study	Ritchie, Lesley J.; Tuokko, Holly [57]	AD, CIND	CSHA	ML	70.2%
Neural networks for longitudinal studies in Alzheimer's disease	Tandon, R. et al [65]	AD, MCI	LAARC	ML	87.0%
Morphological hippocampal markers for automated detection of alzheimer's disease and mild cognitive impairment converters in magnetic resonance images	Ferrarini, L. et al. [46]	AD, MCI	Laboratory of Epidemiology, Neuroimaging, and Telemedicine, at the IRCSS San Giovanni di Dio-FBF in Brescia	ML	80.0%
A comparison of three brain atlases for MCI prediction	Ota, K. et al. [49]	AD, MCI	SEAD-J	ML	77.9%
Multiplexed immunoassay panel identifies novel CSF biomarkers for Alzheimer's disease diagnosis and prognosis	Craig S. et al. [30]	AD, MCI	WU-ADRC	ML	-
Modeling screening, prevention, and delaying of Alzheimer's disease: An early-stage decision analytic model	Furiak, N.M. et al. [73]	AD	-	MS	No Prognostic accuracy
Estimation and validation of a multiattribute model of alzheimer disease progression	Stallard, E. et al. [74]	AD	Predictor's Study (1989-2001) and Predictor's Study 2 (1997-2007)	MS	No Prognostic accuracy
